# Inhibition of the IFN-α JAK/STAT Pathway by MERS-CoV and SARS-CoV-1 Proteins in Human Epithelial Cells

**DOI:** 10.3390/v14040667

**Published:** 2022-03-23

**Authors:** Yamei Zhang, Siobhan Gargan, Fiona M. Roche, Matthew Frieman, Nigel J. Stevenson

**Affiliations:** 1 Viral Immunology Group, School of Biochemistry and Immunology, Trinity Biomedical Sciences Institute, Trinity College Dublin, D02 R590 Dublin, Ireland; yzhang6@tcd.ie (Y.Z.); gargansi@tcd.ie (S.G.); 2 Smurfit Institute of Genetics, Trinity College Dublin, D02 VF25 Dublin, Ireland; fmroche@tcd.ie; 3 Department of Microbiology and Immunology, University of Maryland School of Medicine, Baltimore, MD 21201, USA; mfrieman@som.umaryland.edu; 4 Viral Immunology Group, Royal College of Surgeons in Ireland—Medical University of Bahrain, Adliya 15503, Bahrain

**Keywords:** MERS-CoV, SARS-CoV-1, SARS-CoV-2, interferon-alpha, JAK/STAT (janus kinase/signal transducer and activator of transcription), ISG (interferon stimulated genes), immune evasion

## Abstract

Coronaviruses (CoVs) have caused several global outbreaks with relatively high mortality rates, including Middle East Respiratory Syndrome coronavirus (MERS)-CoV, which emerged in 2012, and Severe Acute Respiratory Syndrome (SARS)-CoV-1, which appeared in 2002. The recent emergence of SARS-CoV-2 highlights the need for immediate and greater understanding of the immune evasion mechanisms used by CoVs. Interferon (IFN)-α is the body’s natural antiviral agent, but its Janus kinase/signal transducer and activators of transcription (JAK/STAT) signalling pathway is often antagonized by viruses, thereby preventing the upregulation of essential IFN stimulated genes (ISGs). Therapeutic IFN-α has disappointingly weak clinical responses in MERS-CoV and SARS-CoV-1 infected patients, indicating that these CoVs inhibit the IFN-α JAK/STAT pathway. Here we show that in lung alveolar A549 epithelial cells expression of MERS-CoV-nsp2 and SARS-CoV-1-nsp14, but not MERS-CoV-nsp5, increased basal levels of total and phosphorylated STAT1 & STAT2 protein, but reduced IFN-α-mediated phosphorylation of STAT1-3 and induction of MxA. While MERS-CoV-nsp2 and SARS-CoV-1-nsp14 similarly increased basal levels of STAT1 and STAT2 in bronchial BEAS-2B epithelial cells, unlike in A549 cells, they did not enhance basal pSTAT1 nor pSTAT2. However, both viral proteins reduced IFN-α-mediated induction of pSTAT1-3 and ISGs (MxA, ISG15 and PKR) in BEAS-2B cells. Furthermore, even though IFN-α-mediated induction of pSTAT1-3 was not affected by MERS-CoV-nsp5 expression in BEAS-2B cells, downstream ISG induction was reduced, revealing that MERS-CoV-nsp5 may use an alternative mechanism to reduce antiviral ISG induction in this cell line. Indeed, we subsequently discovered that all three viral proteins inhibited STAT1 nuclear translocation in BEAS-2B cells, unveiling another layer of inhibition by which these viral proteins suppress responses to Type 1 IFNs. While these observations highlight cell line-specific differences in the immune evasion effects of MERS-CoV and SARS-CoV-1 proteins, they also demonstrate the broad spectrum of immune evasion strategies these deadly coronaviruses use to stunt antiviral responses to Type IFN.

## 1. Introduction

Coronaviruses (CoVs) pose a major global threat to humanity. They are a large class of zoonotic viruses that exist widely in nature and infect vertebrates [1]. They are enveloped, single-stranded, positive-sense RNA viruses. Coronaviruses are the largest RNA genomic virus, with a genome of around 30 kilobases (kb) in length [2]. In 2002, Severe Acute Respiratory Syndrome-Coronavirus-1 (SARS-CoV-1) emerged in Guangdong, China and spread across the world. The epidemic lasted 8 months, resulting in 8089 infections and 774 mortalities [3]. Ten years later, in 2012, Middle East Respiratory Syndrome-Coronavirus (MERS-CoV) emerged in the Middle East and spread across 27 countries, leading to 2567 confirmed cases of infection and 886 deaths; although MERS-CoV appears to have been curtailed, new cases have been reported by the WHO in recent months [4]. Infection with SARS-CoV-1 or MERS-CoV results in severe acute respiratory disease, with symptoms including cough, fever, sore throat, muscle aches, respiratory failure, and diarrhea; with mortality rates of 9.6% and 34.4%, respectively [5,6]. Both of these viruses caused the most severe CoV-associated infections in humans, that is, until the 2019 outbreak and 2020–2021 global pandemic of a novel CoV, SARS-CoV-2 [7], that poses the most threatening international viral challenge of our generation.

Upon infection, the CoV Spike (S) protein binds to specific cell receptors. Angiotensin converting enzyme 2 (ACE2) and dipeptidyl peptidase-4 (DPP4, also known as CD26) are bound by the S proteins of SARS-CoV and MERS-CoV, respectively [8]. The RNA genome is then released into the target cell through endosomal or plasma membrane fusion [9,10]. Pathogen recognition receptors (PRRs), including Toll-like receptors (TLRs) and retinoic acid-inducible gene (RIG)-I-like receptors, have been shown to quickly detect the presence of CoVs [11]. Endosomal TLR3 is critical in detecting SARS-CoV-1 and mounting a protective immune response [12]. Cytoplasmic RIG-I and melanoma differentiation-associated 5 (MDA5), sense double-stranded (ds) viral RNA during transcription and replication, and have been implicated in the detection of SARS-CoV-1 and MERS-CoV and the subsequent production of type 1 Interferon (IFN) [13,14,15]. TLR4 may also provide protection from CoV infection, as mice lacking TLR4 were more susceptible to SARS-CoV-1 infection [12] and SARS-CoV-2-S protein has been shown to interact with TLR4, subsequently inducing pro-inflammatory responses [16,17]. TLR7 recognizes viral single-stranded (ss) RNA and has been shown to detect the presence of CoVs, triggering the induction of type 1 IFNs [18,19,20]. It has been reported that several MERS-CoV and SARS-CoV-1 proteins suppress type 1 IFN induction to evade innate antiviral responses [21,22,23,24]. Type 1 IFNs, such as IFN-α, are essential in clearing viral infection via the Janus kinase/signal transducer and activators of transcription (JAK/STAT) pathway [25]. Type 1 IFN receptor binding promotes the activation of receptor-associated Tyrosine Kinase 2 (Tyk2) and JAK1, which leads to receptor phosphorylation and recruitment of STAT1 and STAT2. Phosphorylated STAT1 and STAT2 dissociate from the receptor and bind IFN regulatory factor (IRF) 9, forming the heterotrimeric IFN-stimulated gene factor (ISGF)3 transcription factor complex. ISGF3 translocates to the nucleus and binds to the IFN-stimulated response element (ISRE), promoting transcription of hundreds of IFN-stimulated genes (ISGs), collectively known as the “interferome” [26,27]. In addition, STAT3 is also activated by type 1 IFN and required in the expression of specific ISGs [28]. These ISGs have wide ranging antiviral capabilities [29].

The effective antiviral role of type I IFNs, especially IFN-α, have been harnessed for the treatment of several viral infections, including hepatitis B virus (HBV) [30], hepatitis C virus (HCV) [31], human papillomavirus (HPV) [32], and human herpes virus (HHV) [33]. Unfortunately, trials with therapeutic IFN-α have shown MERS-CoV to have disappointingly weak clinical responses, with a retrospective study finding that IFN-α2a treatment of MERS-CoV patients did not improve the recovery rate [34]. Others have shown that IFN-α did not effectively inhibit SARS-CoV-1 replication in vitro and presented suboptimal responses in SARS-CoV-1 patients [35,36]. This lack of response to exogenous IFN-α suggests that these CoVs encode antagonists that counteract JAK/STAT signalling. Indeed, the nsp1 of SARS-CoV-1 has been shown to decrease STAT1 activation, and SARS-CoV-1 ORF6 has been shown to sequester STAT1 nuclear import factors (karyopherin alpha 2 and karyopherin beta 1) on the endoplasmic reticulum (ER)/Golgi membrane [37,38]. The SARS-CoV-1 ORF3a has been found to cause ER stress and induce serine phosphorylation-dependent degradation of the IFN-α receptor subunit 1 (IFNAR1) [39]. MERS-CoV ORF4a, 4b and M proteins all inhibit IFN signalling through efficiently blocking ISRE promoter activity [21]. Recent studies of SARS-CoV-2 have determined that nsp5 and nsp14 significantly suppress IFN induction and IFN responses [40,41]. ISRE promoter activity was also reduced by the presence of SARS-CoV-2-nsp2 [41,42].

Even though SARS-CoV-1 and MERS-CoV broke out 20 and 10 years ago, respectively, research progression has failed to fully elucidate the function of all their proteins, with little published data specifically on MERS-CoV-nsp2, MERS-CoV-nsp5, and SARS-CoV-1-nsp14. Moreover, the effect of these three viral proteins on the IFN-α JAK/STAT pathway has not been examined. Therefore, this study sought to examine the impact of MERS-CoV-nsp2, MERS-CoV-nsp5, and SARS-CoV-1-nsp14 upon IFN-α JAK/STAT pathway in two respiratory tract epithelial cell lines and HEK293T cells that are widely used for in vitro molecular analysis.

Here, we report that IFN-α-induced STAT1-3 phosphorylation was stunted by expression of MERS-CoV-nsp2 or SARS-CoV-1-nsp14 in both A549 and BEAS-2B cells. Meanwhile, ISG induction was reduced in the presence of MERS-CoV-nsp2 and SARS-CoV-1-nsp14 in A549 and BEAS-2B cells. In contrast, MERS-CoV-nsp5 did not inhibit pSTAT1-3 nor ISG induction in A549 cells. However, even though MERS-CoV-nsp5 did not inhibit pSTAT1-3 in BEAS-2B cells, it did suppress their ISG induction, suggesting an alternative immune evasion mechanism. Further examination revealed that all three viral proteins inhibited IFN-α-mediated nuclear translocation of STAT1 in BEAS-2B cells. These findings suggest that MERS-CoV-nsp2 and SARS-CoV-1-nsp14 suppress Type 1 IFN responses in a range of epithelial cells, whereby MERS-CoV-nsp5 selectively suppresses these antiviral responses in bronchial BEAS-2B cells. Collectively these discoveries reveal the ability of both of these CoVs to block responses to exogenous IFN-α in human epithelial cells, possibly providing evidence for the ineffectiveness of exogenous IFN-α treatment during CoV infection.

## 2. Materials and Methods

### 2.1. Cell Culture

The alveolar basal epithelial A549 cell line (a kind gift from Dr Kim Roberts, Trinity College Dublin), the bronchial epithelial BEAS-2B cell line (a kind gift from Prof Ultan Power, Queen’s University Belfast), and the human embryonic kidney (HEK)293T cell line, were all cultured in DMEM containing 10% FBS and 1% Penicillin/Streptomycin.

### 2.2. Transfection and Treatment

Cells were transfected with 1 μg DNA constructs encoding HA-tagged MERS-CoV-nsp2, MERS-CoV-nsp5, SARS-CoV-1-nsp14 or the EV control pCAGGS, using Lipofectamine 2000 (Invitrogen, San Diego, CA, USA) at a ratio of 2 µL lipofactamine: 1 µg of DNA. Cells were stimulated with IFN-α-2a (Sigma, St. Louis, MO, USA, SRP4594-100UG) at a concentration of 1000 U/mL, which was diluted in serum free DMEM and further treated for the indicated time periods.

### 2.3. Immunoblotting

Cells were lysed in RIPA buffer (20 mM Tris-HCl pH 7.4, 150 mM NaCl, 1 mM EDTA pH 8, 1% TRITON-X, and 0.5% SDS) supplemented with 1 mM PMSF, 1 mM Na_3_VO_4_, 5 μg/mL leupeptin, and 1 mM DTT and analysed by immunoblotting using antibodies against HA, pSTAT1, STAT1, pSTAT2, STAT2, pSTAT3, STAT3 (Cell Signalling Technology, Danvers, MA, USA) and β-actin (Sigma) and HRP-linked secondary anti-mouse or anti-rabbit antibodies (Invitrogen) and visualised using the Bio-rad ChemiDoc MP imaging system. Blots were analysed using Image Lab software (Bio-rad laboratories, New York, NY, USA).

### 2.4. qRT-PCR

RNA was isolated from cells using TRI Reagent following the manufacturer’s instructions (Sigma). RT-PCR was performed using Sensi-FAST reverse transcriptase (Bioline, Cardiff, UK). qRT-PCR was performed using SYBR-green (Bio-rad) at the following parameters: 95 °C for 15 min, 39 cycles of 95 °C for 30 s, 59 °C for 1 min, and 72 °C for 30 s, using primers specific for the following human genes obtained from Sigma. All genes names, along with forward and reverse primers were summarised in Table 1. Data analysis was carried out using the 2^−∆∆ct^ method. The relative expression of each result was calculated based on expression of the constitutively expressed housekeeping reference gene ribosomal protein 15 (RPS15).

### 2.5. Immunofluorescence Assay

Cells were grown on coverslips and transfected with EV or HA-tagged MERS-CoV-nsp2, SARS-CoV-1-nsp14, or MERS-CoV-nsp5. After 24 h, cells were stimulated for 30 min with IFN-α (1000 U/mL), then fixed at room temperature for 15 min in PBS containing 4% paraformaldehyde (Sigma-Aldrich, St. Louis, MO, USA), before permeabilizing at room temperature for 30 min with 1% Triton X-100 (Sigma-Aldrich) in PBS. Cells were blocked by using 0.5% BSA in PBS for 45 min, followed by overnight incubation with HA (Cell Signalling Technology 3724S 1:800) and STAT1 (Bioscience AHO0832 1:250) primary antibodies. The secondary antibodies, anti-CF™ 568 (Merck, Kenilworth, NJ, USA, SAB4600084 1:100) and anti-Alexa Fluor 647 (MSC 405322 1:1000), were then applied and incubated for 1 h. Cells were washed with PBS three times before using Prolong gold mounting media (contain DAPI) (Thermo Fisher Scientific, Waltham, MA, USA, P36941) to stain nuclei. Images were analysed by using Imaris software. Nuclear translocation of STAT1 in EV control, MERS-CoV-nsp2, SARS-CoV-1-nsp14, and MERS-CoV-nsp5-expressing cells was quantified from three fields of view, collected from three independent experiments. The ratio of nuclear to cytoplasm of STAT1 of each sample was calculated firstly and then compared to IFN-α-treated EV controls, which were normalised to 100%. Scale bar, 20 μm.

### 2.6. Statistical Analysis

Data from repeated experiments were averaged and expressed as the mean ± SEM. Statistical analysis was performed by GraphPad Prism 8 using the Student’s *t*-test or two-way ANOVA analysis. * *p* < 0.05, ** *p* < 0.01, *** *p* < 0.001, **** *p* < 0.0001 (Student’s *t* test and Two-way ANOVA).

## 3. Results

### 3.1. MERS-CoV-nsp2 and SARS-CoV-1-nsp14, but Not MERS-CoV-nsp5, Inhibit IFN-α-Induced pSTAT1-3 in A549 Cells

**Figure 1:**
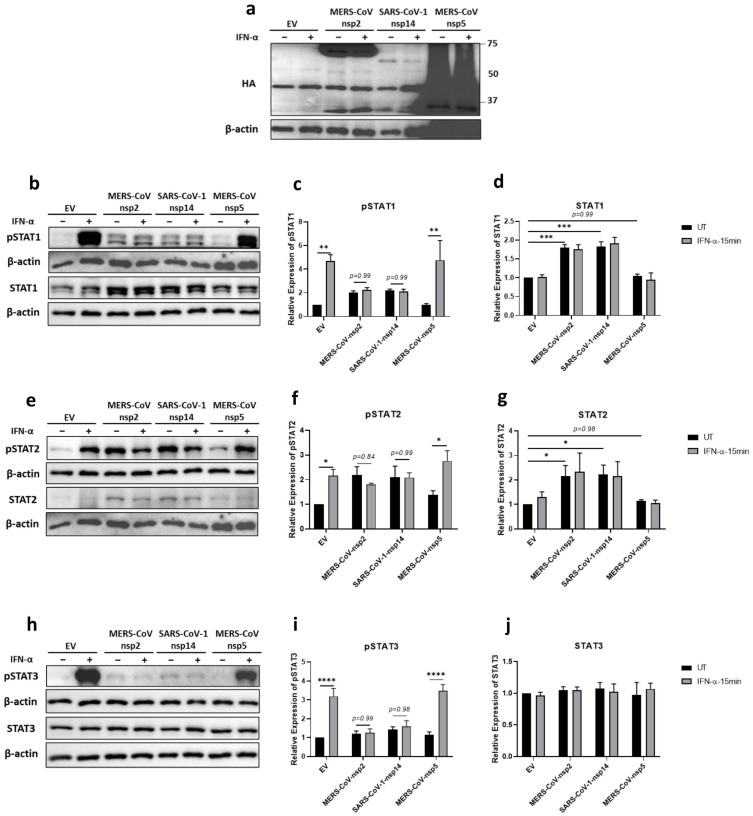
MERS-CoV-nsp2 and SARS-CoV-1-nsp14 expression induce total STAT1 and STAT2 and reduce IFN-α-mediated phosphorylation of STAT1-3 in A549 cells. A549 cells were transfected with Empty Vector (EV), HA-tagged MERS-CoV-nsp2, SARS-CoV-1-nsp14, or MERS-CoV-nsp5. After 24 h, cells were treated with IFN-α (1000 U/mL) for 15 min. Lysates were generated and subjected into immunoblotting with antibodies for (**a**) HA (**b**) pSTAT1 and STAT1, (**e**) pSTAT2 and STAT2, or (**h**) pSTAT3 and STAT3. All blots were also probed with β-actin antibody. (N.B pSTAT1 and STAT2 were probed in one membrane and therefore share the same β-actin, and the pSTAT2 and STAT1 were probed in one membrane and therefore share the same β-actin). Densitometric analysis of (**c**) pSTAT1, (**d**) STAT1, (**f**) pSTAT2, (**g**) STAT2, (**i**) pSTAT3 and (**j**) STAT3 was performed using Image Lab software and values for STATs or phosphorylated STATs were calculated relative to β-actin and compared to the EV transfected UT (untreated) control, which was normalised to 1. All graphs are the mean ± SEM of three independent experiments. * *p* < 0.05, ** *p* < 0.01, *** *p* < 0.001, **** *p* < 0.0001 (Two-way ANOVA).

The modest effect of IFN-α treatment in patients infected with MERS-CoV or SARS-CoV-1 suggested that the IFN-α JAK/STAT signalling pathway was hindered during infection [34,36]. Since STAT1, STAT2, and STAT3 are essential antiviral transcription factors [28,45,46], we firstly analysed the effect of MERS-CoV-nsp2, SARS-CoV-1-nsp14 and MERS-CoV-nsp5 upon their IFN-α-mediated tyrosine phosphorylation. A549 cells were transfected with DNA constructs encoding MERS-CoV-nsp2, SARS-CoV-1-nsp14, MERS-CoV-nsp5, or Empty Vector (EV) control for 24 h, prior to stimulation with IFN-α for 15 min. Expression of all three viral proteins was confirmed by immunoblotting for their HA tags (Figure 1a). Western blotting and densitometric analysis revealed that STAT1 (Figure 1b,d) and STAT2 (Figure 1e,g) protein expression was significantly increased in the presence of MERS-CoV-nsp2 and SARS-CoV-1-nsp14, whereas they had no effect upon STAT3 protein levels (Figure 1h,j). Furthermore, MERS-CoV-nsp5 expression had no effect upon total STAT1 (Figure 1b,d), STAT2 (Figure 1e,g), nor STAT3 (Figure 1h,j) protein levels.

Immunoblotting revealed “basal” phosphorylation of STAT1 and STAT2 upon expression of MERS-CoV-nsp2 and SARS-CoV-1-nsp14 in A549 cells that were untreated with IFN-α (Figure 1b,e); while expression of neither viral protein affected basal pSTAT3 (Figure 1h). pSTAT1 (Figure 1b,c), pSTAT2 (Figure 1e,f), and pSTAT3 (Figure 1h,i) were significantly increased in EV-transfected A549 cells upon stimulation with IFN-α for 15 min; however, this significant increase in pSTAT1-3 was lost in cells expressing MERS-CoV-nsp2 or SARS-CoV-1-nsp14 (Figure 1b,c,e,f,h,i). Unlike MERS-CoV-nsp2 and SARS-CoV-1-nsp14, expression of MERS-CoV-nsp5 did not inhibit IFN-α-mediated phosphorylation of STAT1, STAT2, and STAT3, compared to EV controls (Figure 1b,c,e,f,h,i).

Together, these findings reveal that MERS-CoV-nsp2 and SARS-CoV-1-nsp14, but not MERS-CoV-nsp5, block exogenous IFN-α-mediated phosphorylation of STAT1-3 in A549 epithelial cells, possibly revealing a novel mechanism by which MERS-CoV and SARS-CoV-1 inhibit antiviral responses to Type 1 IFN.

### 3.2. MERS-CoV-nsp2 and SARS-CoV-1-nsp14, but Not MERS-CoV-nsp5, Inhibit IFN-α-Induced pSTAT1-3 in BEAS-2B Cells

Epithelial cells are the first target of respiratory virus infection, however, there are several epithelial cell types distributed throughout the respiratory tract [47]. A549 cells are an alveolar epithelial cell line sourced from a lung cancer, and BEAS-2B cells are a non-tumorigenic, human bronchial epithelial cell [48,49]. Having analsyed the effect of MERS-CoV-nsp2, SARS-CoV-1-nsp14, and MERS-CoV-nsp5 upon IFN signalling in the alveolar A549 cells, we next measured their effect in bronchial BEAS-2B cells.

**Figure 2:**
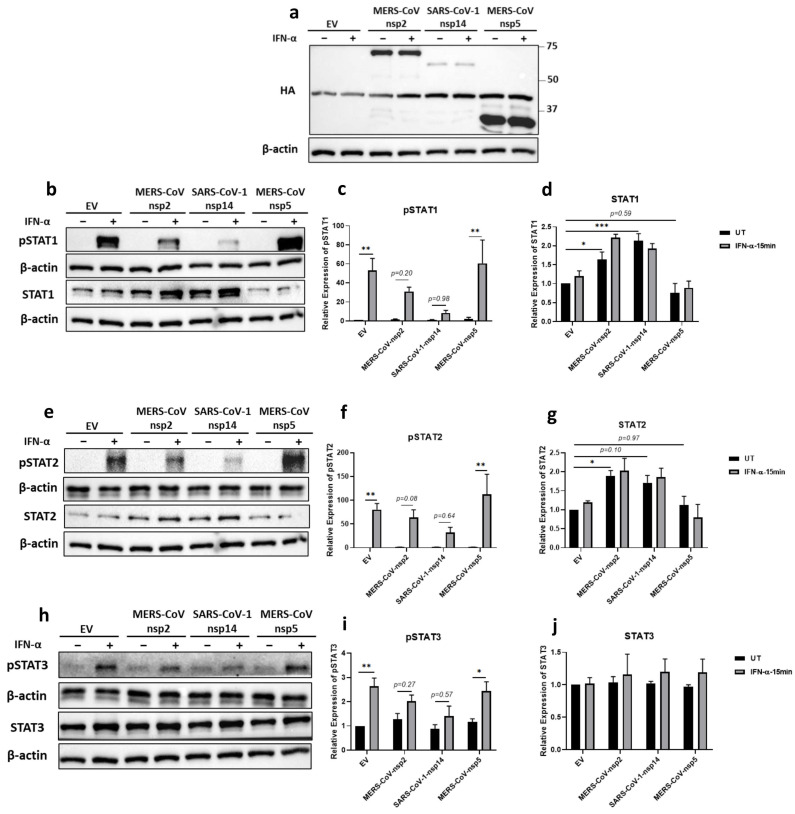
MERS-CoV-nsp2 and SARS-CoV-1-nsp14 expression induce total STAT1 and STAT2 and reduce IFN-α-mediated phosphorylation of STAT1-3 in BEAS-2B cells. BEAS-2B cells were transfected with Empty Vector (EV) or HA-tagged MERS-CoV-nsp2, SARS-CoV-1-nsp14, or MERS-CoV-nsp5. After 24 h, cells were treated with IFN-α (1000 U/mL) for 15 min. Lysates were generated and subjected into immunoblotting with antibodies for (**a**) HA, (**b**) pSTAT1 and STAT1, (**e**) pSTAT2 and STAT2, or (**h**) pSTAT3 and STAT3. All blots were also probed with β-actin antibody. (N.B pSTAT1 and pSTAT2 were probed in one membrane and therefore share the same β-actin, and the STAT2 and STAT1 were probed in one membrane and therefore share the same β-actin). Densitometric analysis of (**c**) pSTAT1, (**d**) STAT1, (**f**) pSTAT2, (**g**) STAT2, (**i**) pSTAT3 and (**j**) STAT3 was performed using Image Lab software, and values for STATs or phosphorylated STATs were calculated relative to β-actin and compared to the EV transfected UT (untreated) control, which was normalised to 1. All graphs are the mean ± SEM of three independent experiments. * *p* < 0.05, ** *p* < 0.01, *** *p* < 0.001 (Two-way ANOVA).

The expression of all three viral proteins was confirmed by immunoblotting for their HA tags (Figure 2a). Similarly to A549 cells, we observed a significant increase in the expression of STAT1 and STAT2, but not STAT3, in BEAS-2B cells, after 24 h transfection of MERS-CoV-nsp2 (Figure 2b,d,e,g,h,j). SARS-CoV-1-nsp14 expression significantly increased STAT1 protein expression (Figure 2b,d), but not STAT3 (Figure 2h,j). Immunoblotting STAT2 protein revealed it to be visually increased upon expression of SARS-CoV-1-nsp14 (Figure 2e); however, while densitometric analysis revealed a 1.7-fold increase compared to EV controls, this was not statistically significant (*p* = 0.1) (Figure 2g).

Unlike MERS-CoV-nsp2 and SARS-CoV-1-nsp14 expression in A549 cells (Figure 1b,e), MERS-CoV-nsp2 and SARS-CoV-1-nsp14 expression in BEAS-2B cells did not induce “basal” pSTAT1 nor pSTAT2 (Figure 2b,e). However, similarly to A549, the statistically significant IFN-α-mediated induction of pSTAT1 (Figure 2b,c), pSTAT2 (Figure 2e,f), and pSTAT3 (Figure 2h,i) in BEAS-2B cells transfected with EV, was lost in cells expressing MERS-CoV-nsp2 or SARS-CoV-1-nsp14. However, as we observed in A549 cells, IFN-α-mediated pSTAT1-3 remained significantly inducible in MERS-CoV-nsp5 expressing BEAS-2B cells (Figure 2b,c,e,f,h,i).

These results reveal that, unlike in A549 cells, MERS-CoV-nsp2 and SARS-CoV-1-nsp14 do not induce basal phosphorylation of STATs in BEAS-2B cells. However, MERS-CoV-nsp2 and SARS-CoV-1-nsp14 (but not MERS-CoV-nsp5) inhibit the ability of exogenous IFN-α to induce significant STAT1-3 phosphorylation in both alveolar (A459) and bronchial epithelial (BEAS-2B) cells, revealing a similar inhibitory pattern in epithelial cells from different regions of the respiratory airways.

### 3.3. MERS-CoV-nsp2, SARS-CoV-1-nsp14 and MERS-CoV-nsp5 Have Little Effect upon the IFN-α JAK/STAT Pathway in HEK293T Cells

**Figure 3:**
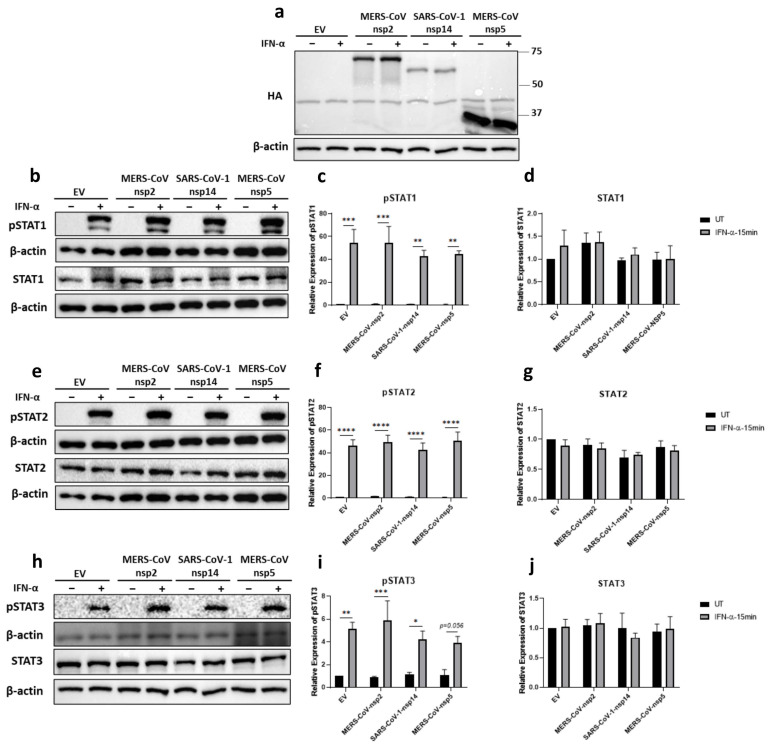
MERS-CoV-nsp2 and SARS-CoV-1-nsp14 expression have no effect on total STAT levels nor their phosphorylation in HEK293T cells. HEK293T were transfected with Empty Vector (EV) or HA-tagged MERS-CoV-nsp2, SARS-CoV-1-nsp14, or MERS-CoV-nsp5. After 24 h, cells were treated with IFN-α (1000 U/mL) for 15 min. Lysates were generated and subjected to immunoblotting with antibodies for (**a**) HA; (**b**) pSTAT1 and STAT1; (**e**) pSTAT2 and STAT2; or (**h**) pSTAT3, STAT3, and HA. All blots were also probed with a β-actin antibody. (N.B pSTAT1 and STAT2 were probed in one membrane and therefore share the same β-actin, and the pSTAT2 and STAT1 were probed in one membrane and therefore share the same β-actin). Densitometric analysis of (**c**) pSTAT1, (**d**) STAT1, (**f**) pSTAT2, (**g**) STAT2, (**i**) pSTAT3 and (**j**) STAT3 was performed using Image Lab software and values for STATs or phosphorylated STATs were calculated relative to β-actin and compared to the EV transfected UT (untreated) control, which was normalised to 1. All graphs are the mean ± SEM of three independent experiments. * *p* < 0.05, ** *p* < 0.01, *** *p* < 0.001, **** *p* < 0.0001 (Two-way ANOVA).

Since HEK293T cells are easily grown and transfected, viral protein transfection studies are often carried out using this cell line. Therefore, we next explored the effect of MERS-CoV-nsp2, MERS-CoV-nsp5, and SARS-CoV-1-nsp14 expression upon the IFN-α JAK/STAT pathway in HEK293T cells, by transfecting them with either of these viral genes or EV for 24 h, before stimulating with IFN-α for 15 min. The expression of all three viral proteins was confirmed by immunoblotting for their HA tags (Figure 3a). Expression of the viral proteins had no effect on protein levels of STAT1 (Figure 3b,d), STAT2 (Figure 3e,g), nor STAT3 (Figure 3h,j). pSTAT1-3 were all significantly induced upon IFN-α treatment (Figure 3b,c,e,f,h,i). Expression of the viral proteins had no effect upon IFN-α-mediated pSTAT2 (Figure 3e,f). While densitometric analysis indicated some variation in the level of pSTAT1 and pSTAT3 phosphorylation upon viral protein expression (Figure 3b,c,h,i), these were minor when compared to the stark effects previously observed in A549 and BEAS-2B cells upon MERS-CoV-nsp2 and SARS-CoV-1-nsp14 (Figure 1 and Figure 2), revealing cell type-dependent variation between kidney (HEK293T) and epithelial (A549 and BEAS-2B) cells lines.

### 3.4. MERS-CoV-nsp2 and SARS-CoV-1-nsp14 Reduce IFN-α-Mediated MxA Induction in A549 Cells

**Figure 4:**
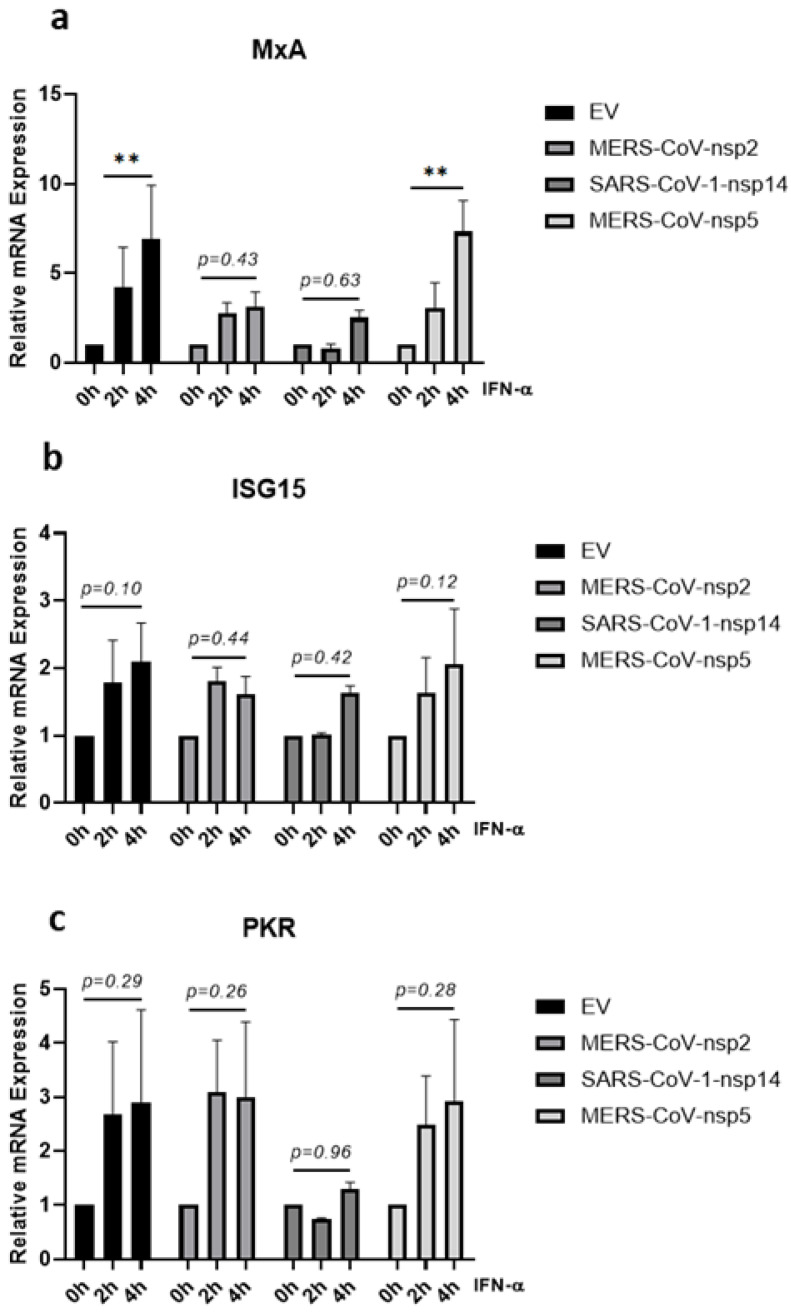
MERS-CoV-nsp2 and SARS-CoV-1-nsp14 expression impair IFN-α-mediated MxA induction in A549 cells. A549 cells were transfected with Empty Vector (EV) or HA-tagged MERS-CoV-nsp2, SARS-CoV-1-nsp14, or MERS-CoV-nsp5. After 24 h, cells were treated for 2 or 4 h with IFN-α (1000 U/mL) before analysing: (**a**) MxA, (**b**) ISG15, and (**c**) PKR mRNA by qRT-PCR. Gene expression was normalised to house-keeping gene RPS15 and IFN-α treated samples were compared to the untreated control (IFN-α 0 h), which was normalised to 1. All graphs are the mean ± SEM of three independent experiments. ** *p* < 0.01 (Two-way ANOVA).

Having observed that a significant increase in IFN-α-mediated STAT1, STAT2, and STAT3 phosphorylation was lost upon expression of MERS-CoV-nsp2 and SARS-CoV-1-nsp14 in A549 cells (Figure 1b,c,e,f,h,i), we next examined if downstream antiviral ISG induction was also affected. A549 cells were transfected with MERS-CoV-nsp2, SARS-CoV-1-nsp14, MERS-CoV-nsp5, or EV for 24 h and stimulated with IFN-α for 0, 2, or 4 h, before measuring mRNA levels of three key ISGs (MxA, ISG15, and PKR) by qRT-PCR. While MERS-CoV-nsp2 expression had no effect upon basal mRNA levels of MxA, ISG15 nor PKR, SARS-CoV-1-nsp14 expression significantly increased basal MxA and PKR mRNA levels (Supplementary Figure S1A–C). MERS-CoV-nsp5 expression reduced basal ISG15 mRNA levels (Supplementary Figure S1B), but had no effect upon basal MxA nor PKR (Supplementary Figure S1A,C). IFN-α treatment did not significantly induce either ISG15 nor PKR mRNA in A549 cells (Figure 4b,c). However, the significant IFN-α-mediated induction of MxA mRNA (in EV controls) was lost upon expression of either MERS-CoV-nsp2 or SARS-CoV-1-nsp14 (Figure 4a). In contrast, induction of MxA mRNA remained statistically significant after 4 h of IFN-α in MERS-CoV-nsp5-expressing cells (Figure 4a). Together these findings suggest that expression of MERS-CoV-nsp2 and SARS-CoV-1-nsp14, but not MERS-CoV-nsp5, suppressed the IFN-α-mediated induction of MxA in A549 cells.

### 3.5. MERS-CoV-nsp2, MERS-CoV-nsp5 and SARS-CoV-1-nsp14 Reduce IFN-α-Mediated MxA, ISG15 and PKR Induction in BEAS-2B Cells

**Figure 5:**
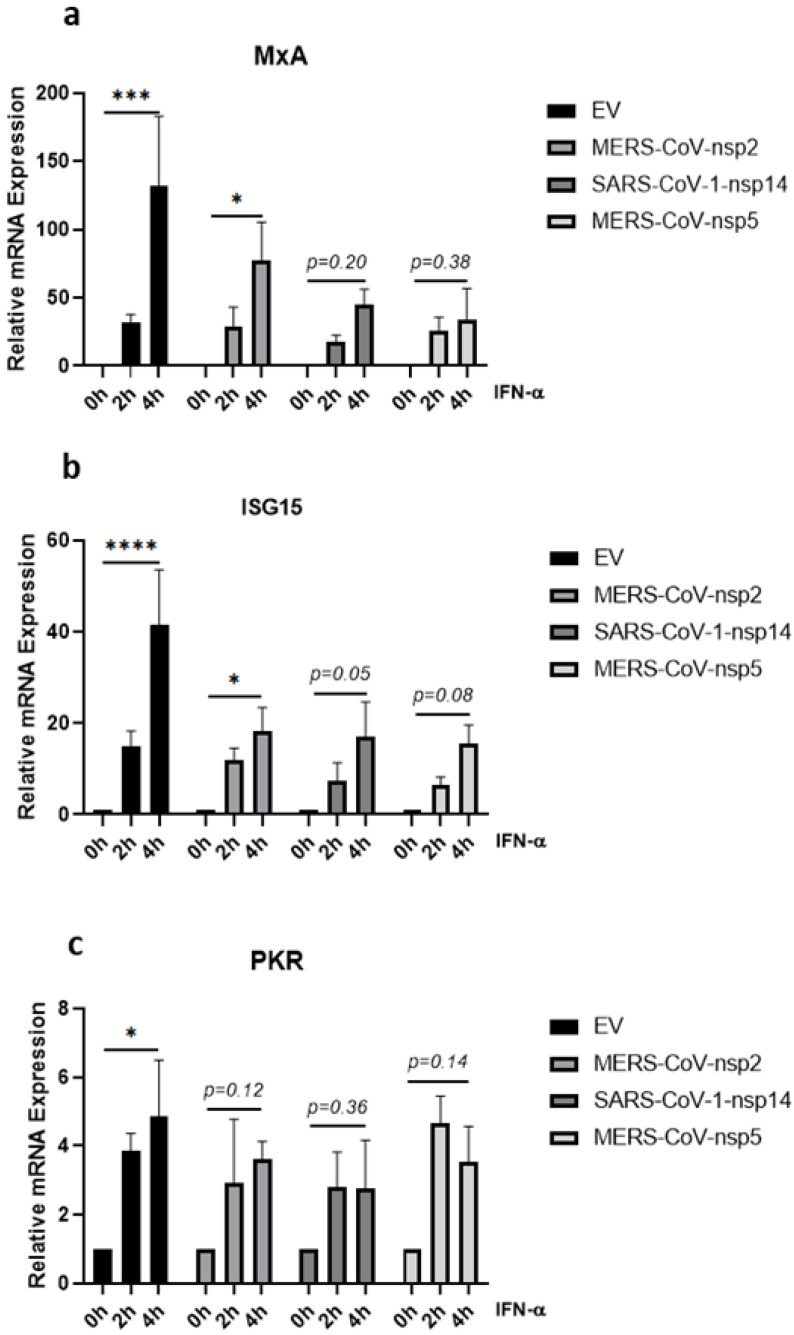
MERS-CoV-nsp2, SARS-CoV-1-nsp14, and MERS-CoV-nsp5 expression impair IFN-α-mediated ISG induction in BEAS-2B cells. BEAS-2B cells were transfected with Empty Vector (EV) or HA-tagged MERS-CoV-nsp2, SARS-CoV-1-nsp14, or MERS-CoV-nsp5. After 24 h, cells were treated with IFN-α (1000 U/mL) for 2 or 4 h before analysing: (**a**) MxA, (**b**) ISG15, and (**c**) PKR mRNA by qRT-PCR. Gene expression was normalised to house-keeping gene RPS15 and IFN-α treated samples were compared to the untreated control (IFN-α 0 h), which was normalised to 1. All graphs are the mean ± SEM of three independent experiments. * *p* < 0.05, *** *p* < 0.001, **** *p* < 0.0001 (Two-way ANOVA).

Given that the significant increase of IFN-α-induced phosphorylation of STAT1-3 was lost in BEAS-2B cells expressing MERS-CoV-nsp2 and SARS-CoV-1-nsp14 (Figure 2b,c,e,f,h,i), we next investigated whether the expression of MERS-CoV-nsp2 and SARS-CoV-1-nsp14 affected downstream IFN-α-induced ISGs in BEAS-2B cells. MERS-CoV-nsp2, SARS-CoV-1-nsp14, MERS-CoV-nsp5, or EV control were transfected into BEAS-2B cells for 24 h, before stimulating with IFN-α for 0, 2, or 4 h and measuring mRNA levels of MxA, ISG15, and PKR by qRT-PCR. Basal MxA mRNA levels were induced by SARS-CoV-1-nsp14 (Supplementary Figure S2A) and basal mRNA level of PKR was decreased by MERS-CoV-nsp5 (Supplementary Figure S2C). Four hours of IFN-α treatment significantly induced MxA, ISG15, and PKR mRNA in EV control cells; however, this significance was lost in SARS-CoV-1-nsp14 or MERS-CoV-nsp5-expressing cells (Figure 5a–c). MERS-CoV-nsp2 expression blocked the significant induction of PKR (Figure 5c) and reduced the significance of MxA and ISG15 induction (Figure 5a,b). Taken together, these results suggest that the previously observed MERS-CoV-nsp2 and SARS-CoV-nsp14 inhibition of IFN-α-mediated STAT phosphorylation (Figure 2b,c,e,f,h,i) may attenuate downstream ISG expression in BEAS-2B cells. However, having previously observed that MERS-CoV-nsp5 did not block STAT1-3 phosphorylation in BEAS-2B cells (Figure 2b,c,e,f,h,i), we hypothesised that this viral protein inhibited IFN-α-induced MxA, ISG15, and PKR via an alternative mechanism.

### 3.6. MERS-CoV-nsp2, MERS-CoV-nsp5 and SARS-CoV-1-nsp14 Inhibit Nuclear Translocation of STAT1 in BEAS-2B Cells

**Figure 6:**
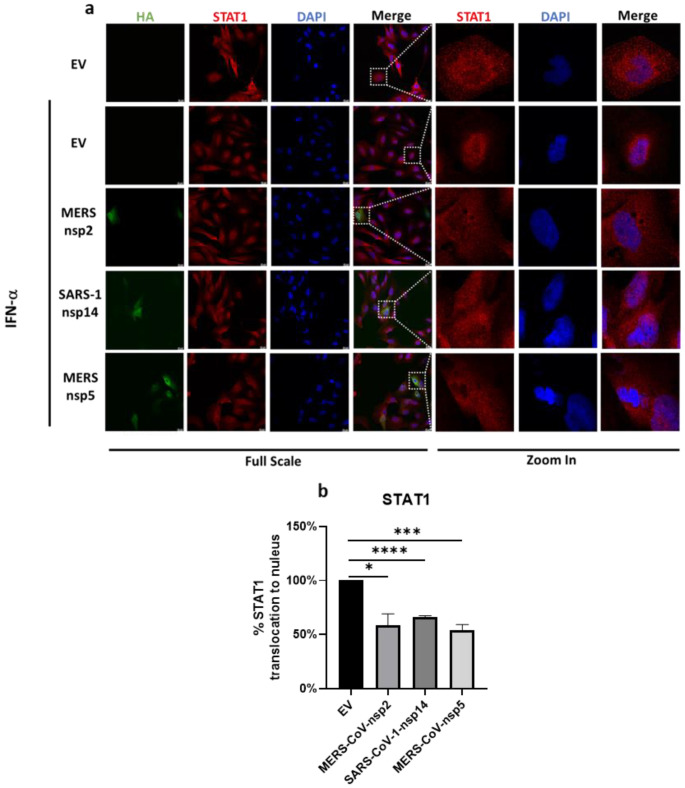
MERS-CoV-nsp2, SARS-CoV-1-nsp14, and MERS-CoV-nsp5 expression block IFN-α-mediated STAT1 nuclear translocation of BEAS-2B cells. (**a**) Confocal microscopy images of BEAS-2B cells transfected with Empty Vector (EV) or HA-tagged MERS-CoV-nsp2, SARS-CoV-1-nsp14, or MERS-CoV-nsp5. After 24 h, cells were stimulated for 30 min with IFN-α (1000 U/mL) then fixed and permeabilised. Anti-HA and anti-STAT1 were probed as primary antibodies, and anti-CF™ 568 and anti-Alexa Fluor 647 were probed as secondary antibodies to monitor HA expression and STAT1 subcellular localisation. Nuclei were stained by DAPI. Each full scaled image was then zoomed in for presenting single transfected cells. (**b**) Quantification of nuclear translocation of STAT1 in control, MERS-CoV-nsp2, SARS-CoV-1-nsp14, and MERS-CoV-nsp5-positive cells from three fields of view on each coverslip collected from three independent experiments. The level of STAT1 translocation to nucleus for IFN-α-treated EV was normalised to 100%. Scale bar, 20 μm. All graphs are the mean ± SEM of three independent experiments * *p* < 0.05, *** *p* < 0.01, **** *p* < 0.0001 (Student’s *t* test).

Since MERS-CoV-nsp5 expression did not suppress the phosphorylation of STAT1-3 (Figure 2b,c,e,f,h,i), but did reduce IFN-α-mediated ISG induction of bronchial BEAS-2B epithelial cells (Figure 5a–c), we next explored if it could suppress ISG induction via a blockade of STAT1 nuclear translocation. To investigate this, we next transfected MERS-CoV-nsp5, MERS-CoV-nsp2, SARS-CoV-1-nsp14, or EV control into BEAS-2B cells for 24 h and stimulated with IFN-α for 30 min, before analysing the localisation of STAT1 by confocal microscopy. Expressions of all three HA-tagged viral protein (green) and STAT1 protein (red) were monitored with the described primary and fluorescent antibodies. Nuclei was stained using DAPI (blue). To present an individual cell, a section of the 1× image was magnified 16 times (Figure 6a). For immunofluorescence analysis, the mean fluorescence intensity of STAT1 for the single cell was measured and then the ratio of cytoplasmic to nuclear STAT1 was quantified in EV, MERS-CoV-nsp2, SARS-CoV-1-nsp14, and MERS-CoV-nsp5-expressing cells. The level of STAT1 nuclear translocation in IFN-α-treated EV-transfected cells was normalised to 100% and all relative STAT1 nuclear translocation percentages for MERS-CoV-nsp2, SARS-CoV-1-nsp14, and MERS-CoV-nsp5 were plotted on graphs (Figure 6b). Our immunofluorescence analysis showed that “normal” IFN-α-induced STAT1 nuclear translocation of EV-transfected cells (Figure 6a,b) and was significantly reduced upon expression of MERS-CoV-nsp5, MERS-CoV-nsp2 or SARS-CoV-1-nsp14 (Figure 6a,b). While our previous results suggest that MERS-CoV-nsp2 and SARS-CoV-1-nsp14 reduce ISG induction via suppression of STAT1-3 phosphorylation (Figure 2b,c,e,f,h,i), these confocal microscopy results suggest that all three viral proteins inhibit IFN-α-mediated ISG induction by suppressing STAT1 nuclear translocation of bronchial BEAS-2B epithelial cells.

## 4. Discussion

IFN-α signalling is essential for early anti-viral innate immune responses and its induction leads to the up-regulation of hundreds of ISGs. However, many viruses have evolved mechanisms to suppress the IFN-α JAK/STAT pathway. Here we investigated the effect of several non-structural proteins (from MERS-CoV and SARS-CoV-1), upon the IFN-α pathway in epithelial cells. We found that nsp2 from MERS-CoV and nsp14 from SARS-CoV-1 enhanced protein expression of STAT1 and STAT2, but not STAT3, in both A549 and BEAS-2B epithelial cells. In the absence of exogenous Type 1 IFN, MERS-CoV-nsp2 and SARS-CoV-1-nsp14 also induced basal STAT1 and STAT2 tyrosine phosphorylation in A549 cells (but not BEAS-2B cells), suggesting that these viral proteins induce IFNs and/or other cytokines, which may act in a paracrine/autocrine fashion and induce and activate STATs. Indeed, increased cytokine expression is a common feature of both MERS-CoV and SARS-CoV-1 infection [50] and differences between these two epithelial cell lines have previously been observed in response to other viruses. In fact, when compared to BEAS-2B cells, A549 cells produced higher cytokine levels upon respiratory syncytial virus or influenza A virus infection, further supporting our hypothesis that upon expression of MERS-CoV-nsp2 and SARS-CoV-1-nsp14, basal STAT and pSTATs observed in A549 cells, might be as a result of increased cytokine secretion [51,52].

The JAK/STAT pathway plays an essential role in eliminating viruses [6,53], therefore, it is not surprising that STAT protein expression and activation are targeted by these pathogens. Indeed, the Hepatitis B virus (HBV) HBX protein has been shown to interact with JAK1 and activate downstream phosphorylation of STATs [54]. Similarly, v-abl from Abelsom Murine-leukaemia Virus (A-MuLV) interacts with JAK1 and JAK3, also leading to enhanced STAT phosphorylation [55]. The EBV SM protein also enhanced expression of STAT1 mRNA and protein and upregulated ISG expression in B cells [56]. Hepatitis C Virus (HCV) core, NS5B, and p7 proteins have all been shown to regulate STAT3 phosphorylation, which, in the case of HCV-p7, induces SOCS3, thus suppressing proinflammatory TNF-α responses [57,58,59]. Porcine reproductive and respiratory syndrome virus (PRRSV) and EBV have also been shown to induce serine phosphorylation of STAT1 [60,61], with constitutive activation of STAT1 restricting IFN-stimulated STAT1–DNA binding [61]. Recently, STAT2 was also shown to play a dual role in SARS-CoV-2 hamster model infection; whereby STAT2 drove severe lung injury, due to elevated proinflammatory cytokine expression, but also restricted systemic virus dissemination, clearly showing a significant effect of another CoV upon the JAK/STAT pathway [62].

In our current study, we found that the exogenous addition of IFN-α to A549 and BEAS-2B epithelial cells, expressing MERS-CoV-nsp2 and SARS-CoV-1-nsp14, did not induce normal levels of STAT1-3 phosphorylation. This contrasted with HEK293T embryonic kidney cells, where IFN-α-induced STAT1-3 phosphorylation was not affected by either viral protein, identifying differential responses to MERS-CoV and SARS-CoV-1 proteins between human respiratory epithelial and kidney cells. Furthermore, while our over-expression studies did not specifically require CoV cell receptors, DDP4 and ACE2 expression levels are important for infection studies. A549 and HEK293T cells both lack ACE2 and DPP4 expression [63,64,65,66,67], whereby BEAS-2B cells express ACE2 and can thus be readily infected with either SARS-CoV-1 or SARS-CoV-2 [68,69]. While there is little known about DDP4 expression in BEAS-2B cells, a protein atlas database shows DPP4 is moderately expressed in the human bronchus [70], suggesting that BEAS-2B cells could also be susceptible to MERS-CoV infection. Since ACE2 has been revealed as an ISG [71], dysregulation of the IFN-α JAK/STAT pathway might also reduce ACE2 levels, thus inhibiting CoV infection. Type-I IFN signalling was also dysregulated in SARS-CoV-2-infected-human epithelial Calu-3 and Caco-2 cell lines, but as with our findings, this dysregulation was not observed in HEK293T cells [72], again identifying cell-type-specific differences.

The decrease of IFN-α-induced pSTAT1-3 by MERS-CoV-nsp2 and SARS-CoV-1-nsp14 in A549 cells and BEAS-2B cells clearly underlined the ability of these viral proteins to inhibit IFN-α JAK/STAT signalling. The anti-viral activity of STAT1 & STAT2 makes them primary targets for viruses such as RSV, HIV, and HCV [43,73,74]. Even though the anti-viral activity of STAT3 is less clearly defined, its expression has been shown to be indispensable for the IFN-α-mediated upregulation of a specific ISG subset, including MxA and ISG15 [28]. However, several viruses, including mumps, HCV, and HIV, have been shown to target STAT3 and block its anti-viral activity [43,74,75], indicating that our observed CoV-mediated reduction in pSTAT3 may also be responsible for reduced ISG induction. A recent genomics-based study identified differential regulation of STAT3 in two highly pathogenic strains of MERS-CoV (MERS-CoV Eng 1 and MERS-CoV SA 1); as a result, changes in STAT3 activity were speculated to result in altered pro-inflammatory gene expression in human airway Calu-3 cells [76], suggesting that STAT3 is closely related to MERS-CoV pathogenesis. Having shown that MERS-CoV-nsp2 inhibited IFN-α-induced phosphorylation of STAT3 in A549 and BEAS-2B cells, our results further support a role for STAT3 in the anti-viral Type I IFN response during MERS-CoV infection. Our findings are also in agreement with a previous study showing decreased STAT3 phosphorylation in SARS-CoV-1-infected Vero E6 cells [77]; but we may have revealed a specific role for SARS-CoV-1-nsp14, as a suppressor in the pSTAT3 in A549 and BEAS-2B cells.

Type I IFNs stimulate cells to upregulate the expression of ISGs, which inhibit viral infection [53]. In our study we investigated the effect of MERS-CoV-nsp2, MERS-CoV-nsp5, and SARS-CoV-1-nsp14 on several well-characterised anti-viral ISGs (MxA, PKR and ISG15). While these ISGs have several actions, MxA is well known to prevent the endocytic trafficking of viral particles into the cell [78]; PKR prevents viral protein translation [79] and ISG15 inhibits viral transcription, translation, and egress from the cell [29]. This spectrum of anti-viral effects highlights the importance of limiting ISG expression and effectiveness by viruses; for example, HIV-Vif protein has been shown to block IFN-α-induced ISG15 induction and HCV to attenuate IFN-α-induced OAS1 upregulation [43,80]. In this study, we found that significant IFN-α-induced upregulation of MxA was lost in A549 cells expressing either SARS-CoV-1-nsp14 or MERS-CoV-nsp2. However, since we did not observe significant induction of ISG15 nor PKR mRNA in EV control A549 cells, it was impossible to measure any statistical changes between EV and viral protein-expressing cells. One of our previous studies, analysing the effect of IFN-α and Ribavirin upon ISG induction, also showed an expression difference between MxA and other ISGs. We observed an increase in IFN-α & ribavirin-mediated MxA in Huh7 cells, whereas, PKR, OAS, and CXCL10 were not induced, which not only highlights the complexity of specific ISG expression levels, but, combined with our current results, possibly reveals the importance of MxA in combating viral infection [81]. The significant IFN-α-mediated upregulation of all three ISGs in EV-control BEAS-2B cells was lost upon SARS-CoV-1-nsp14 and MERS-CoV-nsp5 expression. While MERS-CoV-nsp2 expression reduced the induction of all three ISGs, PKR was the only ISG to see a statically significant loss. As IFN-α-induced ISG15 induction has been shown to correlate with STAT3 expression [28], the attenuated ISG15 induction in MERS-CoV-nsp2 and SARS-CoV-1-nsp14-expressing BEAS-2B cells may be linked to their decrease in STAT3 phosphorylation. Taken together, these results provide clues to how MERS-CoV and SARS-CoV-1 individual proteins blocked anti-viral ISG induction in epithelial cells, making them an optimal environment for CoV infection and replication.

We found that MERS-CoV-nsp5 expression had no effect on total- nor phopho-STAT1-3 protein levels, but still attenuated IFN-α-mediated ISGs induction, indicating that it regulated the JAK/STAT pathway through a different mechanism than MERS-CoV-nsp2 and SARS-CoV-1-nsp14. Since SARS-CoV-2-nsp5 has been reported to impair the IFN-α-mediated induction of anti-viral ISGs through blocking the nuclear translocation of STAT1 [40], we investigated if MERS-CoV-nsp5 could also be using this immune evasion mechanism. Indeed, consistent with this study, we found that MERS-CoV-nsp5 also reduced the nuclear translocation of STAT1, possibly accounting for the inhibition of IFN-α-mediated ISG induction. Meanwhile, we also found MERS-CoV-nsp2 and SARS-CoV-1-nsp14 inhibited STAT1 nuclear translocation in BEAS-2B cells; together these results reveal that MERS-CoV-nsp2 and SARS-CoV-1-nsp14 target the IFN-JAK/STAT pathway by suppressing both IFN-α-mediated STAT1-3 phosphorylation and STAT1 nuclear translocation, which may result in our observed reduction of downstream ISGs. Similarly, SARS-CoV-2-N and SARS-CoV-2-nsp5 proteins antagonize type I IFN signalling, by both suppressing phosphorylation and nuclear translocation of STAT1 and STAT2 [40,82], highlighting that CoVs may have developed multiple strategies to restrict the anti-viral IFN-α JAK/STAT pathway.

Having observed three deadly CoVs to emerge over the past 20 years, it is clear that we need novel therapeutic approaches for their treatment. IFN-alphacon-1 (synthetic IFN-α), in combination with steroids, has shown partially protective effects in the treatment of SARS-CoV-1 patients [83]. However, other studies failed to see the benefit of IFN-α treatment [36], suggesting that the JAK/STAT pathway is blocked. Clinical trials investigating lopinavir-ritonavir and IFN-β1b combination therapy for MERS-CoV [84] and IFN-β (in combination with other anti-viral treatments), for treatment of SARS-CoV-2 [85,86], are ongoing. Indeed, pre-treatment with IFN-α has shown some protective effects against SARS-CoV-2 infection, and SARS-CoV-2 has been shown to be more sensitive to IFN-α, when compared with SARS-CoV-1 in Vero cells [42,87]. Overall, the results of our study indicate that MERS-CoV-nsp2, MERS-CoV-nsp5, and SARS-CoV-1-nsp14 inhibit the IFN-α JAK/STAT pathway, which may also provide insight into the functional effects of homologous SARS-CoV-2 proteins and even future emergent CoVs. Indeed, the suppressive effects of MERS-CoV-nsp2, MERS-CoV-nsp5, and SARS-CoV-nsp14 upon the IFN-α JAK/STAT pathway, may also explain the previously observed therapeutic failure of Type I IFN. Therefore, it may be beneficial for future studies to focus on restoring IFN-α responsiveness, as a therapeutic approach to existing and future CoV infections.

## Figures and Tables

**Table 1:** qRT-PCR primers sequences.

Gene Name	Forward Primer Sequence	Reverse Primer Sequence	Ref.
*RPS15* (NM_001308226.2) [Housekeeping Reference Gene]	CGGACCAAAGCGATCTCTTC	CGCACTGTACAGCTGCATCA	[43]
*MxA* (NM_001178046.3)	GGTGGTGGTCCCCAGTAATG	ACCACGTCCACAACCTTGTCT	[43]
*ISG15* (NM_005101.4)	TCCTGCTGGTGGTGGACAA	TTGTTATTCCTCACCAGGATGCT	[43]
*PKR* (NM_002759.4)	TCTACGCTTTGGGGCTAA	GCCATCCCGTAGGTCTGT	[44]

## Data Availability

The original contributions presented in the study are included in the article/Supplementary Materials; further inquiries can be directed to the corresponding authors.

## Supplementary Materials

The following supporting information can be downloaded at: https://www.mdpi.com/article/10.3390/v14040667/s1, Figure S1. Basal expression of ISGs in EV, MERS-CoV-nsp2, SARS-CoV-1-nsp14, and MERS-CoV-nsp5-transfected A549 cells. A549 cells were transfected with Empty Vector (EV), HA-tagged MERS-CoV-nsp2, SARS-CoV-1-nsp14, or MERS-CoV-nsp5. After 24 h, cells were isolated for total RNA before analysing: (**A**) MxA, (**B**) ISG15, and (**C**) PKR mRNAs by qRT-PCR. Gene expression was normalised to house-keeping gene RPS15 and compared to the EV control, which were normalised to 1. All graphs are the mean ± SEM of three independent experiments. * *p* < 0.05, *** *p* < 0.001 (Student’s *t* test); Figure S2. Basal expression of ISGs in EV, MERS-CoV-nsp2, SARS-CoV-1-nsp14, and MERS-CoV-nsp5-transfected BEAS-2B cells. BEAS-2B cells were transfected with Empty Vector (EV), HA-tagged MERS-CoV-nsp2, SARS-CoV-1-nsp14, or MERS-CoV-nsp5. After 24 h, cells were isolated for total RNA before analysing: (**A**) MxA, (**B**) ISG15, and (**C**) PKR mRNAs by qRT-PCR. Gene expression was normalised to house-keeping gene RPS15 and compared to the EV control, which were normalised to 1. All graphs are the mean ± SEM of three independent experiments. * *p* < 0.05, ** *p* < 0.01 (Student’s *t* test).
