# Supplementary material for: Inhibition of the IFN-α JAK/STAT Pathway by MERS-CoV and SARS-CoV-1 Proteins in Human Epithelial Cells
Source: Viruses. 2022 Mar 23;14(4):667. doi: 10.3390/v14040667 (PMC9032603; doi:10.3390/v14040667)
Supplement: Supplementary file 1 [file viruses-14-00667-s001.zip › viruses-1611376-supplementary.pdf]

viruses

MDPI

Article

Inhibition of the IFN-α JAK/STAT pathway by MERS-CoV and SARS-CoV-1 proteins in human epithelial cells

Yamei Zhang [1], Siobhan Gargan [1], Fiona M. Roche [2], Matthew Frieman [3] and Nigel J. Stevenson [1,4,*]

1 Viral Immunology Group, School of Biochemistry and Immunology, Trinity Biomedical Sciences Institute, Trinity College Dublin, D02 R590 Dublin, Ireland; yzhang6@tcd.ie (Y.Z.); gargansi@tcd.ie (S.G.)
2 Smurfit Institute of Genetics, Trinity College Dublin, D02 VF25 Dublin, Ireland; fmroche@tcd.ie
3 Department of Microbiology and Immunology, University of Maryland School of Medicine, Baltimore, MD 21201, USA; mfrieman@som.umaryland.edu
4 Viral Immunology Group, Royal College of Surgeons in Ireland—Medical University of Bahrain, Adliya 15503, Bahrain
* Correspondence: n.stevenson@tcd.ie

Supplementary Materials:

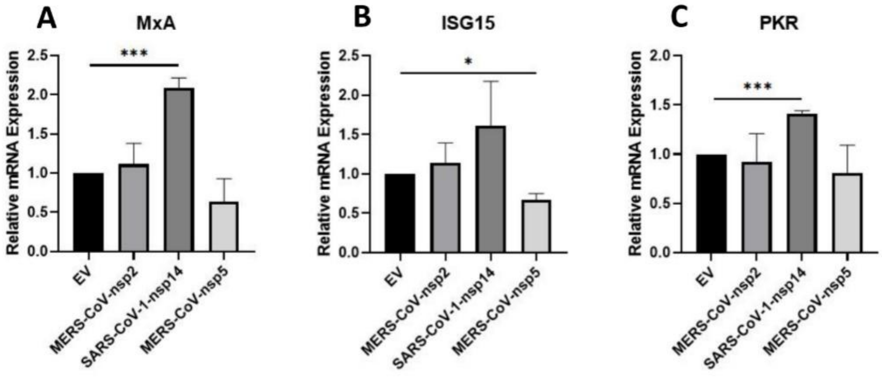

Figure S1. Basal expression of ISGs in EV, MERS-CoV-nsp2, SARS-CoV-1-nsp14 and MERS-CoV-nsp5-transfected A549 cells. A549 cells were transfected with Empty Vector (EV) or HA-tagged MERS-CoV-nsp2, SARS-CoV-1-nsp14 or MERS-CoV-nsp5. After 24 hours, cells were isolated for total RNA before analysing (**A**) MxA (**B**) ISG15 (**C**) PKR mRNAs by qRT-PCR. Gene expression was normalised to house-keeping gene RPS15 and compared to the EV control, which were normalised to 1. All graphs are the mean ± SEM of three independent experiments. **p<0.05, ***p<0.001 (Student's t test)*

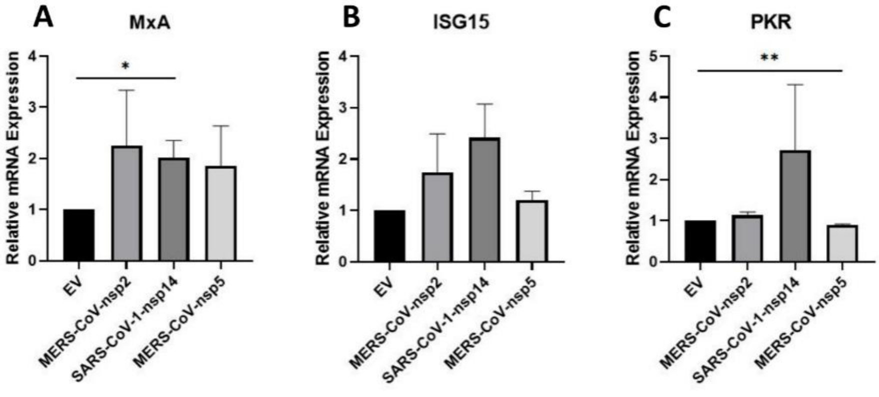

Figure S2. Basal expression of ISGs in EV, MERS-CoV-nsp2, SARS-CoV-1-nsp14 and MERS-CoV-nsp5-transfected BEAS-2B cells. BEAS-2B cells were transfected with Empty Vector (EV) or HA-tagged MERS-CoV-nsp2, SARS-CoV-1-nsp14 or MERS-CoV-nsp5. After 24 hours, cells were isolated for total RNA before analysing (**A**) MxA (**B**) ISG15 (**C**) PKR mRNAs by qRT-PCR. Gene expression was normalised to house-keeping gene RPS15 and compared to the EV control, which were normalised to 1. All graphs are the mean ± SEM of three independent experiments. **p<0.05, **p<0.01 (Student's t test)*
